# Predicting the Intensity of Psychedelic-Induced Mystical and Challenging Experience in a Healthy Population: An Exploratory Post-Hoc Analysis

**DOI:** 10.2147/NDT.S426193

**Published:** 2023-10-05

**Authors:** Kwonmok Ko, Ben Carter, Anthony J Cleare, James J Rucker

**Affiliations:** 1 Centre for Affective Disorders, Department of Psychological Medicine, Institute of Psychiatry, Psychology & Neuroscience, King’s College London, London, SE5 8AF, UK; 2 Department of Biostatistics and Health Informatics, Institute of Psychiatry, Psychology and Neuroscience, King’s College London, London, SE5 8AF, UK; 3 South London and Maudsley NHS Foundation Trust, Bethlem Royal Hospital, Beckenham, BR3 3BX, UK

**Keywords:** mystical experience, challenging experience, psilocybin, psychedelic therapy

## Abstract

**Introduction:**

In psychedelic therapy, mystical as well as challenging experience may influence therapeutic outcome. However, predictors of such experience have not been sufficiently established. Determining predictors of their intensity is, therefore, potentially beneficial in targeting psilocybin therapy for depression.

**Methods:**

In a post hoc data analysis of a Phase 1, randomised, double-blind, placebo-controlled, between-groups clinical trial, dosage, personality traits, affect, and individual data were analysed as possible clinical predictors. Eighty-nine healthy volunteers were randomised to receive a single dose of placebo, 10 mg of psilocybin, or 25 mg of psilocybin. ANOVA was used to analyse the relationship between dosage and mystical and/or challenging experience, and correlation analysis for all other variables.

**Results:**

The intensity of both mystical and challenging experience was strongly associated with higher dosage. Age was negatively correlated with intensity of challenging experience. Correlation between identified personality traits and either mystical or challenging experience was minimal, with the exception of positive correlation between neuroticism and challenging experience at higher dose. Neither positive nor negative affect indicated correlation with the intensity of either type of experience.

**Discussion:**

A limitation of this study is its post hoc, exploratory design; recommendations for further research are provided.

## Introduction

Psychedelics are psychoactive drugs currently under active clinical research, notably in treatment-resistant depression.^1^,^2^ During the acute phase of the drug’s action, subjective effects commonly include mystical and/or challenging experiences.^3^ The predictive aspects of said experiences have not yet been fully explored.

Mystical experience [ME] was identified by Stace^4^ to include ineffability, deeply felt positive mood, paradoxicality, sacredness, noetic quality, and transcendence of time and space. The ability to induce ME by psychedelics may mediate clinical improvement.^5^ The correlation between therapeutic efficacy and the presence, as well as intensity, of ME have been identified in multiple systematic reviews of psychedelic therapies for psychiatric disorders including substance use disorders, depression, and cancer-related distress.^6–8^ Long-term positive changes as predicted by ME have been demonstrated in multiple clinical trials including treatment-resistant depression,^9^ depression and anxiety in patients with life-threatening cancer,^10^ and studies measuring self-reported “personal growth”.^11^,^12^

Oceanic boundlessness [OBN] is one measure of ME and is defined as perception of self without boundaries.^13^ The 5 Dimensions Altered State of Consciousness Rating Scale [5D-ASC]^14^ measures oceanic boundlessness [OBN], with subdimensions of unity, spiritual experience, bliss, disembodiment, and insightfulness. Liechti et al^15^ demonstrated that OBN, on the 5D-ASC, has a Pearson correlation of 0.93 with the Mystical Experience Questionnaire [MEQ].

Psychedelic-induced challenging experiences include physical reactions such as headaches, increased heart rate, blood pressure, and respiration rate;^16^ psychological reactions include transient distress, anxiety, and dread of ego dissolution [DED].^17^ The 5D-ASC^14^ includes a DED dimension, with sub-dimensions of anxiety and impaired control or cognition, in which higher scores indicate a more challenging experience.^9^,^16^ In an online survey study of 1993 participants,^18^ duration of psilocybin-induced challenging experience has been negatively associated with sustained benefits on well-being in a multiple regression analysis; further, the intensity of said experience had a significant positive correlation with increased life satisfaction, spiritual significance, and attribution of enduring personal meaning. In contrast, evidence from clinical trials in depressed patients suggests that the presence of challenging experience compromises the positive effects of psychedelic therapy.^9^,^19^

Mystical and challenging experiences have not yet been established as predictors of therapeutic outcome. If efficacy of psychedelic therapy can be predicted positively by mystical experience, and negatively by challenging experience, the prediction of such may help to optimise outcome in psychedelic therapy. Potential predictors include personality traits, affect, and dosage; other potential factors include individual factors, such as age, biological sex, and previous psychedelic experience, each subsequently discussed.

### Personality

The 5-factor model of personality, as developed by Goldberg^20^ and Digman,^21^ consists of 5 traits including openness, neuroticism, conscientiousness, agreeableness, and extraversion. Extraversion, agreeableness, and conscientiousness have not been analysed as predictors for mystical and/or challenging experience to the best of authors’ knowledge; openness and neuroticism have been significantly correlated with mystical and challenging experience.

In a double-blind, placebo-controlled, between-subject trial of ayahuasca, Smigielski et al^22^ established openness as a predictor of OBN, as well as of all mysticism dimensions (η^2^ = 0.12–0.18), on the Hood Mysticism Scale.^23^ A contrasting result was found by MacLean et al^24^ who analysed data from two previous double-blind trials^5^,^25^ and found that screening levels of Openness did not correlate significantly with mystical experience as measured by Mystical Experience Questionnaire [MEQ] (*r* = 0.12, *p* = 0.41). This was further supported by a prospective online survey study^26^ which also found that openness to new experience had no effect on acute psychedelic experience.

Analysis of 2 online surveys of those who have taken psilocybin^27^ showed that higher scores of neuroticism were associated with higher scores on the Challenging Experience Questionnaire [CEQ]. “Emotional stability”, defined as the inverse of neuroticism, associated negatively with the CEQ, while neuroticism associated positively. Conversely, in a retrospective study conducted by Studerus et al^28^ which pooled data from 23 controlled psilocybin experiments of 261 healthy volunteers, no statistically significant association was found between neuroticism and challenging experience. It is worth noting that Studerus et al showed absorption trait to be directly correlated with mystical experience; however, data available for this post-hoc study did not include this trait.

### Affect

Affect as a predictor of mystical and/or challenging experience has not been studied sufficiently to date, to the authors’ knowledge. In the limited background literature, Studerus et al^28^ found emotional excitability prior to drug administration to be predictive of both mystical and challenging experience. It is therefore reasonable to assume that affect would have bearing on the outcome of a psychedelic experience.^29^ According to the Positive and Negative Affect Scale [PANAS],^30^ positive affects are defined as being interested, excited, strong, alert, enthusiastic, proud, inspired, determined, attentive, and active. Affects including being distressed, upset, guilty, hostile, irritable, ashamed, nervous, jittery, afraid, and scared are defined as negative.

### Dosage

Dosage has been studied in relation to the intensity of psychedelic-induced ME in previous trials.^18^,^28^,^31^,^32^ Garcia-Romeu et al^32^ in particular demonstrated that 13 out of 42 psilocybin sessions resulted in complete ME (≥0.6 on related subscales of States of Consciousness Questionnaire), 10 (77%) of which were induced during high-dose (30mg/70kg), and the remaining 3 (23%) at moderate (20mg/70kg). Carbonaro et al^18^ demonstrated that dosage was related to challenging experience; however, while duration of difficulty was negatively associated with sustained benefits to well-being, degree of difficulty had a positive association. Additionally, most participants (84%) reported to have ultimately benefited from said experience. The evidence for correlation between dosage and intensity is not yet conclusive.

### Individual Factors

Factors such as age, biological sex, and prior psychedelic experience are often measured in clinical trials but have rarely been analysed as predictors for either mystical or challenging experience.

Studerus et al^28^ found age to be positively associated with the mystical experience of blissful state, and negatively associated with challenging experiences of anxiety, impaired control and cognition, and difficulty of the experience itself, the latter of which was also supported by Carbonaro et al.^18^ In addition, age was negatively associated with dread in a retrospective survey study of 143 participants.^33^ A large-scale (n=3364) study^34^ using data from Global Drug Survey 2020 also found younger age to be associated with longer duration and higher occurrence of negative outcomes in association with recreational psychedelic administration.

In a systematic review of 14 studies by Aday et al,^35^ most studies indicated no sex difference. Naïveté to psychedelic experience was positively correlated with challenging experience to include feelings of being overwhelmed, resistant and distressed.^36^ Studerus et al^28^ also noted a correlation between psychedelic naïveté and intensity in most dimensions of ME, although statistical significance was only established for a few.

It must be noted in all of the above studies that there is a broad interindividual and methodological variation; therefore, the background of this study lacks consistency.

### Aims and Hypotheses

#### Aims

In this analysis of a previously published clinical trial, we aimed to explore predictors of mystical and challenging experiences in healthy volunteers given psilocybin in a clinical setting.

#### Hypotheses

1. Psilocybin dose will positively predict the intensity of both mystical and challenging experience.
2. Increased age will positively predict the intensity of psilocybin-induced mystical experience and negatively predict the intensity of challenging experience.
3. Absence of prior psilocybin experience will positively predict both the intensity of psilocybin-induced mystical and challenging experience.
4. Biological sex will not predict the intensity of either mystical or challenging experience.
5. The degree of trait openness will positively predict the intensity of mystical experience and negatively predict the intensity of challenging experience. 
6. The degree of trait neuroticism will negatively predict the intensity of mystical experience and positively predict the intensity of challenging experience. 
7. The degree of trait extraversion, agreeableness, and conscientiousness will not predict the intensity of mystical experience nor predict the intensity of challenging experience.
8. The degree of positive affect will positively predict the intensity of mystical experience and negatively predict the intensity of challenging experience.
9. The degree of negative affect will negatively predict the intensity of mystical experience and positively predict the intensity of challenging experience.

## Materials and Methods

Data were extracted from the clinical trial of Rucker et al^37^ (EudraCT number: 2018-000978-30). The authors of this analysis were granted access to relevant data by the Sponsor of the trial (COMPASS Pathfinder Ltd, a subsidiary of COMPASS Pathways Plc.). This was a phase 1, randomised, double-blind, placebo-controlled, between-groups clinical trial in which 89 healthy volunteers were randomised to receive a single dose of placebo, 10 mg of psilocybin, or 25 mg of psilocybin, with 12 weeks of follow-up. The psilocybin used in this study was “COMP360”, which is a proprietary pharmaceutical-grade synthetic psilocybin formulation, optimised for stability and purity, developed by the sponsor, COMPASS Pathfinder Ltd. The primary objectives of the trial were to assess the effects of psilocybin on emotional and cognitive processing in an exploratory design.

### Participants

The inclusion criteria were participants between the ages of 18 and 65 at screening; ability to complete the outcome assessment tools without assistance; and able to speak English. Exclusion criteria were current or past history of mental illness or first-degree relatives with history, and current or recent psychiatric medication; current or history of substance dependence (excluding nicotine); medical history including cardiovascular, diabetes, seizure disorder, or current or recent pregnancy or intension of same; and, enrolment in any other clinical trial. See Rucker et al^37^ for further detail.

### Materials

The data for this analysis was drawn from three of the instruments used in the original trial.

The NEO-Five Factor Inventory [NEO-FFI]^38^ consists of 60 questions assessing individuals on five dimensions of personality to include extraversion, agreeableness, conscientiousness, neuroticism, and openness to experience.

The 5-Dimension Altered States of Consciousness Scale [5D-ASC]^14^ includes 94 questions that determine state of oceanic boundlessness, dread of ego dissolution, vigilance reduction, visionary restructuralization, and auditory alterations.

The Positive and Negative Affect Schedule [PANAS],^30^ which was previously discussed, was applied in its entirety.

### Design

The predictor variables assessed were personality, measured by NEO-FFI; affect, measured by PANAS; individual data including biological sex, age, and prior psilocybin experience; and dosage of psilocybin. The outcome variables were mystical and challenging experiences, as measured by 5D-ASC subdimensions OBN and DED, respectively.

### Procedure

Subjects completed baseline assessments (PANAS and NEO-FFI), one day prior to dosing. The dosing session with psilocybin or placebo was 6 to 8 hours in duration. Immediately following the dosing session, the 5D-ASC was administered. See Rucker et al^37^ for further detail.

### Statistical Analysis

This study was exploratory and not powered to formally detect statistical significance. IBM SPSS Statistics (Version 28)^39^ was used to run the statistical analysis. A Pearson correlation was fitted to investigate the potential association between personality traits and affect, and outcome variables of OBN and DED, stratified by dosage group. For individual data, the same analysis was applied, but they were analysed according to control and treatment groups. Analysis of Variance [ANOVA] was used to investigate the relationship between predictor variable of dosage and outcome variables of OBN and DED, comparing the two mean difference [MD] of OBN and DED score between placebo and both 10mg and 25mg pairwise comparison. The MD will be presented alongside the 95% confidence intervals [95% CI]. Due to the exploratory post-hoc nature of the study, no correlation was made for multiple testing for the two pairwise comparisons.

### Ethics and Approvals

The original study^37^ was a Clinical Trial of an Investigational Medicine Product (CTIMP) authorised by the Medicines and Healthcare Products Regulatory Agency (MHRA) and the National Research Ethics Committee (NREC) in the UK. The Sponsor was COMPASS Pathfinder Ltd. All participants signed an informed consent form prior to eligibility screening. The study was conducted in accordance with the International Conference on Harmonisation guidelines for Good Clinical Practice, the Declaration of Helsinki, and was approved by the London-Brent Research Ethics Committee (reference number: 18/LO/0731).

## Results

Eighty-nine participants were randomised among 25 mg psilocybin (n=30), 10 mg psilocybin (n=30), and placebo (n=29) dosing groups. Participants were stratified by sex and age in the randomisation (18–35 years old; >35 years old). There were four withdrawals, all from the placebo group, which were excluded from the Modified Intent-to-Treat Population. Subjects’ ages ranged from 20 to 59 (mean=36.1, SD=9.06). There were 48 males (53.9%) and 41 females (46.1%). Thirty-three (37.1%) had prior psilocybin experience, which represents 24.1% from the placebo group (n=7) compared to 36.7% (n=11) and 50% (n=15) from the 25mg and 10mg psilocybin groups, respectively. The current study analysed data from all 85 participants who completed the trial.

### Dosage

**Figure 1:**
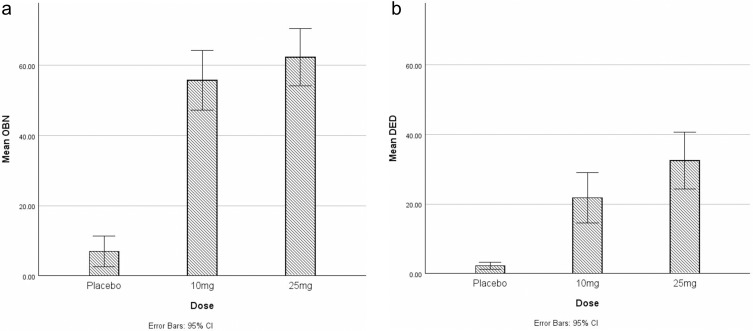
(**a**) Mean oceanic boundlessness (OBN) score by dosage. (**b**) Mean dread of ego dissolution (DED) score by dosage.

Regarding OBN, the mean difference between 25mg and placebo (55.34, *p*<0.001, 95% CI: 42.58 to 68.10) was greater than the mean difference between 10mg and placebo (48.74, *p*<0.001, 95% CI: 35.98 to 61.50) [See Figure 1a]. Concerning DED, the mean difference between 25mg and placebo (30.20, *p*<0.001, 95% CI: 18.77 to 41.63) was greater than the mean difference between 10mg and placebo (19.45, *p*<0.001, 95% CI: 8.02 to 30.88) [See Figure 1b]. The residuals were approximately normally distributed with a zero mean and constant variance.

### Individual Data

**Figure 2:**
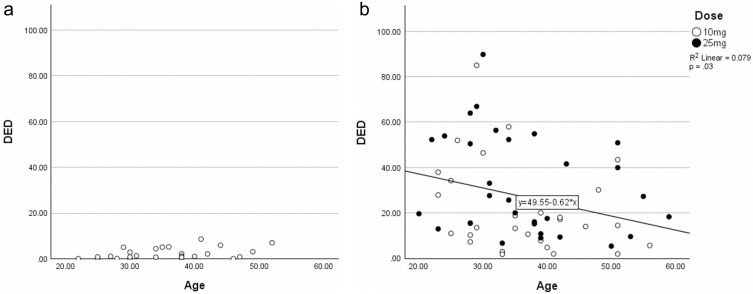
(**a**) Scatterplots of age and dread of ego dissolution (DED) scores in placebo group. (**b**) Scatterplots of age and dread of ego dissolution (DED) scores in dosage groups.

Among three individual data predictor variables (age, biological sex, and prior psilocybin experience), only age showed correlation with DED, which was nominally significant. In a single group which included both 10 and 25 mg, higher age correlated with lower DED score (*r* = −0.28, 95% CI: −0.50 to −0.03, *p* = 0.03) [See Figure 2a and b].

### Personality/Affect

With the exception of a positive correlation between neuroticism and DED at 25 mg dosage (*r* = 0.38, *p* < 0.039), neuroticism did not demonstrate significant correlation with OBN or DED. [See relevant scatterplots in Supplementary File 1].

The other personality traits – openness, agreeableness, extraversion, and conscientiousness – did not demonstrate any significant correlation with OBN or DED. [See relevant scatterplots in Supplementary Files 2–5].

Neither positive nor negative affect demonstrated significant correlation with OBN or DED. [See relevant scatterplots in Supplementary Files 6 and 7].

## Discussion

The original hypotheses of this post hoc analysis predicted that in a healthy adult trial population, personality traits, affect, individual factors of age and prior psilocybin experience, and dosage would be associated with intensity of psilocybin-induced mystical and/or challenging experience, while the individual factor of biological sex would show no correlation with either.

For the variable of dosage, data indicated that the intensity of both mystical and challenging experience is associated with higher dosage, supporting the hypothesis. This is in keeping with previous studies of this nature.^18^,^31^,^32^ However, the increase in intensity of mystical experience between dosage levels is less than that of challenging experience. This implies that higher dosages of psilocybin may correspond with increasingly challenging experience, without necessarily implying a corresponding increase in mystical experience. Duration rather than intensity of challenging experience may be a more clinically meaningful measure,^18^ although this was not captured in our data; for example, if a challenging experience is intense but of short duration, it is likely to be more easily tolerated. Challenging experience may provide a catalyst for new insights and/or emotional breakthrough that leads to clinical benefits, however more granular data about the nature and degree of individual challenging experiences than was available here is likely needed to unpick this hypothesis further.

The results supported hypotheses for both biological sex and age. Biological sex was not correlated with mystical or challenging experience, similar to the findings in the systematic review of Aday et al.^35^ Age was negatively correlated with intensity of challenging experience, similar to the finding of Studerus et al,^28^ Kopra et al,^34^ and Russ et al,^33^ as we had hypothesised. This suggests that age may be a protective factor against psilocybin-induced challenging experience in healthy subjects. This likely reflects the influence of psychological or biological factors associated with increasing age, such as the development of better coping skills for negative or challenging experience, rather than age itself. Whilst this may have some relevance to recreational use of psilocybin mushrooms in a healthy population, the clinical implications are minimal. We cannot extrapolate these findings to patients and, even if age was found to be a useful clinical predictor, it would be difficult ethically to justify the exclusion of patients from a treatment on the basis of age, unless a serious safety signal was found in future trials.

In contradiction to our hypothesis and studies of psychedelic naïveté by Studerus et al^28^ and McCartney et al,^36^ prior psilocybin experience did not show significant correlation with intensity of mystical or challenging experience. Our analysis may have been affected by the fact that those with psilocybin experience within one year prior to the study were excluded from participation.

The hypothesis for neuroticism was partially supported; at higher dosage (25mg), a positive correlation with intensity of challenging experience was demonstrated, similar to the findings of Barrett et al^27^ but in contrast to the study of Studerus et al.^28^ Neuroticism, as measured by the NEO-FFI, may be a useful measure to identify those individuals at higher risk of challenging experience, although we cannot reasonably extrapolate from this sample into a clinical patient population.

The personality trait of openness showed no correlation with either mystical or challenging experience, in keeping with the findings of Haijen et al,^26^ but in contrast to the findings of MacLean et al^24^ and Smigielski et al.^22^ The evidence in this area remains inconclusive and still warrants further study. The other personality traits – extraversion, conscientiousness, and agreeableness – were not significant predictors of mystical or challenging experience, thus supporting the hypothesis.

The hypotheses regarding affect were unsupported. We find no evidence that affect as measured by the PANAS predicts the experience of OBN or DED.^29^ While the data available for this study did not include the factor of emotional excitability as referenced by Studerus et al,^28^ this would be an area for future study.

As this was a post hoc analysis, our hypotheses were not pre-registered, and we may reasonably be criticised for selective analysis. Similarly, since the original trial was not designed to meet the needs of our analysis, we may have been underpowered to explore our hypotheses. Excessive variance may, in this case, lead to false-positive or false-negative findings. However, the advantage of this exploratory analysis is that it does allow an estimation of numbers needed for a more statistically robust study. Since repeating a clinical study specifically to explore these hypotheses is likely not to be cost-efficient, we might seek to explore these ideas more economically using national surveys on drug use and health data, as in Kopra et al.^34^ Another option is citizen science initiatives, by which data concerning psychedelic use under participants’ own supervision can be collected for analysis with results being shared with these participants.^40^,^41^ This also provides an opportunity to explore duration of both mystical and challenging experience as a criterion variable, in comparison to intensity. Additionally, re-examination of personality traits and affect variables, as well as replication of the findings on dosage and age, with larger data sets might provide further insight. A study of these variables with both healthy and clinical population for comparison is also recommended as it could provide new insight for therapeutic application.

## Conclusion

In this post hoc study of an existing clinical trial, we found evidence of psilocybin dosage as a predictor of both mystical and challenging experience. Additionally, we found indication of age and the personality trait of neuroticism as predictive for intensity of mystical and/or challenging experience. With the advent of broader methods of data collection, such as national surveys or citizen science, replication of this study with larger samples may be possible. Whilst our findings currently have limited clinical application, they may have relevance to healthy populations. Further study of these variables with both healthy and clinical populations is warranted.
